# Therapeutic Implications of Caffeic Acid in Cancer and Neurological Diseases

**DOI:** 10.3389/fonc.2022.860508

**Published:** 2022-03-10

**Authors:** Manzar Alam, Sarfraz Ahmed, Abdelbaset Mohamed Elasbali, Mohd Adnan, Shoaib Alam, Md. Imtaiyaz Hassan, Visweswara Rao Pasupuleti

**Affiliations:** ^1^Centre for Interdisciplinary Research in Basic Sciences, Jamia Millia Islamia, New Delhi, India; ^2^Department of Biosciences, Jamia Millia Islamia, New Delhi, India; ^3^Department of Clinical Laboratory Science, College of Applied Sciences-Qurayyat, Jouf University, Sakakah, Saudi Arabia; ^4^Department of Biology, College of Science, University of Hail, Hail, Saudi Arabia; ^5^Department of Biotechnology, Jamia Millia Islamia, New Delhi, India; ^6^Department of Biomedical Sciences and Therapeutics, Faculty of Medicine & Health Sciences, University Malaysia Sabah, Kota Kinabalu, Malaysia; ^7^Department of Biochemistry, Faculty of Medicine and Health Sciences, Abdurrab University, Pekanbaru, Indonesia; ^8^Centre for International Collaboration and Research, Reva University, Rukmini Knowledge Park, Kattigenahalli, Bangalore, India

**Keywords:** caffeic acid, clinical trials, diabetic neuropathy, inflammatory diseases, inhibitors, targeted therapy

## Abstract

Caffeic acid (CA) is found abundantly in fruits, vegetables, tea, coffee, oils, and more. CA and its derivatives have been used for many centuries due to their natural healing and medicinal properties. CA possesses various biological and pharmacological activities, including antioxidant, anti-inflammatory, anticancer, and neuroprotective effects. The potential therapeutic effects of CA are mediated *via* repression and inhibition of transcription and growth factors. CA possesses potential anticancer and neuroprotective effects in human cell cultures and animal models. However, the biomolecular interactions and pathways of CA have been described highlighting the target binding proteins and signaling molecules. The current review focuses on CA’s chemical, physical, and pharmacological properties, including antioxidant, anti-inflammatory, anticancer, and neuroprotective effects. We further described CA’s characteristics and therapeutic potential and its future directions.

## Introduction

Caffeic acid (CA), one of the most common phenolic acids (PhA), frequently occurs in fruits (1), grains (2), and dietary supplements (3) for human consumption as simple esters with quinic acid or saccharides. It is found in fruits, tea, coffee, oil, spices, and vegetables. CA is isolated and purified from green and roasted coffee sources. Food coming directly from plants is a rich source of phytochemicals. They are considered biologically active compounds, though with negligible nutritional values. Studies reveal their major role in chronic disease prevention. These plant compounds are antioxidants, pharmacological agents, detoxifying/cleansing agents, and dietary fiber (4, 5). CA (3,4-dihydroxycinnamic acid) is a phytochemical that prevents several human diseases, and it is manufactured through the hydrolysis of chlorogenic acid (6, 7).

CA illustrated a wide range of chemical and pharmacological properties (4). It is orally bioavailable due to its anti-inflammatory, antioxidant, and anticancer bioactivities (8–10) and immunomodulatory and neuroprotective activities (11–13). CA has antitumor effects against several human cancers (14, 15). It is an excellent antioxidant and anti-inflammatory agent. CA exhibits the medicinal properties of reducing oxidative stress and significantly inhibits damage to DNA by free radicals (16, 17). CA is known for preventing cancer cell growth by inhibiting the HDM histone demethylase oncoprotein gene during cancer progression (18), and explains neuroprotective results against amyloid-β–mediated neurotoxicity *via* blocking calcium influx and tau phosphorylation. It may contribute to preventing neurodegeneration and brain injury (19, 20).

CA has established its importance as an effective 5-lipooxygenase inhibitor and has revealed its capacity for downregulating IL-6, IL-1β, and NF-κB in the inflammatory response (21, 22). CA drastically blocks STAT3 action and this, in turn, down-triggers HIF-1α action. It has the promising inhibitors of STAT3 and represses cancer angiogenesis *via* blocking the action of STAT3 and the expression of VEGF and HIF-1α (14). CA blocked ERKs phosphorylation, NF-κB, and AP-1 transactivation. However, CA targets MEK1 and TOPK for repressing tumor metastasis and neoplastic cell transformation (15). It frankly interrelated with ERK1/2 and blocked ERK1/2 actions *in vitro* (23).

This review focuses on the effects of CA, such as antioxidant, anti-inflammatory, anticancer, and neuroprotective effects. The potential of CA in cancer and neurological diseases with emphasis on clinical trials has been summarized. This review also describes CA’s features and therapeutic potential and its implications in cancer and neurological disease treatment and management.

## Biosynthesis of Phenolic Acids

Higher plants accumulate and synthesize a broad diversity of phenolic compounds that confer defense against the assaults of free radicals created through the process of photosynthesis, as well as against tissue damages (24). Phenolic compounds offer defense against diseases by regulating cellular mechanisms at different levels, such as enzyme inhibition, protein phosphorylation, and alteration of gene expression (25). Phenolics are essential ingredients in plants, commonly found in fruits, cereals, legumes, and vegetables. In plants, they have a role in a broad range of processes, including growth, pigmentation, reproduction, as well as resistance to pathogens. They are categorized into five classes: flavonoids, coumarins, tannins, phenolic acids, and stilbenes (26, 27). However, phenolics are secondary metabolites primarily created in plants from shikimic acid *via* phenylpropanoid signaling (26). They are created as an effect of the break of lignin and cell wall polymers in vascular plants and the like via the production of monolignol signaling (26, 27). PhA are the furthermore classified into cinnamic acids and benzoic acids. They are present in free appearances or conjugated with ethers, esters, as well as other molecules (26–29). The structural motifs needed for the antitumor action of phenolic compounds comprise the aromatic ring, unsaturated, substituted chains, and the position and number of free hydroxyl groups (26). Phenolics are admired for their effectiveness as medicinal compounds in the treatment and management of numerous diseases, including cancer and neurodegenerative diseases. Their antidiabetic function is induced by the modulation of glucose metabolism (27, 30).

## Chemical and Physical Properties of Caffeic Acid

The defensive effect of CA on the human body is described because of its antioxidant functions, which are endorsed to its chemical structure. However, the antioxidant functions of CA are linked with the existence of two hydroxyl groups at its aromatic ring. The structure of CA has a phenol ring with OH at positions 3 and 4 of the ring and a hydrocarbon chain at position 1 with an acid group. The chemical structure characteristics of CA make it an efficient metal-reducing agent. It is prone to auto-oxidation and oxidation by biological agents. The catechol group with an unsaturated carboxylic acid chain interacts with the oxidizing radicals. Chemically, it is cinnamic acid. Its chemical structure comprises a phenyl ring with hydroxyl groups at the 3rd and 4th positions. More commonly in transform, the cis form also exists (31). Thus, ultimately this structural uniqueness imparts anticancer properties (32, 33).

The CA derivative CAPE has a similar structure but with an additional phenylmethyl ester group. However, the chemical and physical properties of the two vary (34, 35). CA is a phenolic compound consequential from the secondary metabolism of plant results (36, 37). For example, olives, fruits, potatoes, coffee beans, carrots, and propolis are the major hydroxycinnamic acid utilized in human diets (38–40). CA is present in yellow crystals that turn alkaline solution from yellow to orange. It is sparingly soluble in cold water and highly soluble in hot water and cold alcohol. It has a small weighted compound with zero formal charges (41, 42).

## Bioavailability and Pharmacokinetics of Caffeic Acid

CA is absorbed in the GI tract by MA transporters (43, 44) and by the transepithelial flux to a slighter extent (45). Gut microbiota is linked to the metabolism of CA. Actually, under an anaerobic situation, CA undertakes decarboxylation that is passed out through the bacteria with the analogous product [3-(3- hydroxyphenyl)-propionic acid] exhibiting better antioxidant action in contrast to CA (46). Once absorbed, CA undertakes widespread metabolic transformations in the kidneys and liver (47, 48). The small intestine is the probable location of the cleavage of feruoylquinic acids liberating CA and ferulic acid, the metabolism of CA to its 3- and 4-O-sulfates, and the methylation of CA for forming isoferulic acid and its corresponding 3-O-sulfation and glucuronidation, since the colon is possibly the location of the metabolism of CA to dihydrocaffeic acid that is promoted to be metabolized for dihydro-isoferulic acid. CA is emitted mainly by the urine, which calculates urinary secretion between 5.9% and 27% (32, 49, 50). Accordingly, the pharmacokinetic procedure starts with the ingestion of CA, inward in the stomach, following a little part that is absorbed. Hence, in the colon, the microbial esterases slice the ester piece of CA; in its free appearance, it is absorbed through the intestinal mucosa (51). The transmembrane run of CA into intestinal cells takes place *via* active transport mediated through the MCT (51).

The highest plasma concentration of the compound has been detected merely 1 h after ingestion of foods, including coffee. Subsequently, plasma concentration is quickly reduced, requiring reiterated doses every 2 h to sustain high concentrations (52–54). Chemical flexibility and modifiability of CA affected its phenylpropanoid scaffold for becoming a usually utilized pattern for improving novel derivatives with increased pharmacokinetic functions, enhanced bioactivity, and superior safety summary (55). However, studies have shown that hepatic metabolism of CA and DHCA occurs when O-methylation, dehydrogenation, hydrogenation, and GSH conjugation take place. Detoxification for CA and DHCA comes from the O-methylation pathway, whereas the toxification occurs *via* P450 catalyzation to form o-quinones (56, 57). Humans have CA metabolites, mainly glucuronides of meta-coumaric acid and meta-hydroxyhippuric acid. The derivative of CA, CAPE, synthesized by combining CA and phenethyl alcohol in the ratio of 1:5 at room temperature along with the condensing agent DCC, has been proven to have therapeutic potential (35, 58). CAPE arrests the growth of HL-60 cells in leukemia. Different IC_50_ values of 1.0, 0.5, and 1.5 µM inhibit the DNA, RNA, and protein expression, respectively (59).

## Biological Mechanism of Action of Caffeic Acid

### Oxidative Stress

Reactive oxygen species (ROS) are linked with numerous cellular functions, including cell differentiation, proliferation, angiogenesis, and apoptosis. ROS are the main signaling molecule that participates in an essential function in developing inflammatory diseases such as cancer and neurodegenerative diseases. Oxidative stress and chronic inflammation are the most crucial factors engaged in the progression of about 15–20% of cancers worldwide (60). The constant inflammatory cell employment, frequent production of ROS, and pro-inflammatory mediators, which persisted the proliferation of genomically unbalanced cells, help the neoplastic transformation and eventually result in cancer metastasis and invasion (61). The inequity between the production and clearance of ROS/RNS aids the progression of the tumor chiefly *via* stimulating genomic instability. CA blocks lipoxygenase action and represses LPO (62, 63). CA entirely inhibits ROS production and the xanthine oxidase system (62). CA might enhance the cytotoxic activities of M1 macrophages and block cancer growth; inhibitory action on TAMs can be induced by its antioxidative action. However, the anticancer action of CA has been the effect of the synergistic actions of diverse mechanisms through which CA performs on proliferation, survival, angiogenesis, and immunomodulation (64–66). CA competently inhibited the incidence of TAMs and noticeably repressed tumorigenesis in mouse tumor models. Hence, targeting TAMs *via* antioxidants might be a potentially effective method for tumor treatment (64).

The amount of such ROS-mediated injuries was revealed to be more widespread in the areas of the brain where Aβ is rich (67, 68). The antioxidant defense in the AD brain was found to repeatedly reduce as the disease progressed (69–71). Mitochondrial dysfunction is the main cause of ROS-induced pathologies far too frequent in AD (72). Thus, drugs targeting several mechanisms of ROS/oxidative stress-induced cellular injuries could have therapeutic potential for AD. Hence, a study (73) has examined the anti-AD potential of CA in an *in vitro* acrolein-mediated toxicity model utilizing HT22 mouse hippocampal cells. However, the study has revealed that pretreatment of cells with CA drastically reduced the acrolein-mediated neurotoxicity, ROS gathering, and GSH reduction. The acrolein-mediated MAPKs and GSK3β/Akt signalings have been altered *via* CA, recommending their probable mechanisms by such results. The improvement of memory *via* CA (10–50 mg/kg) in the global cerebral ischemia-reperfusion damage in the rat is partly because of antioxidant results, as a reduced MDA and considerable SOD activities have been detected to be reduced in the hippocampus’s subsequent treatment with CA (22). The defensive result of CA against spatial learning and memory role failure *via* chronic aluminum excess has been revealed to be accompanied by the reverse of the reduced SOD actions and enhanced MDA contents (21). However, the association between cognitive decline and oxidative stress is fine as demonstrated in most of the work by researchers (74) who utilized streptozotocin-mediated investigational dementia in rats like an AD model.

### Antioxidant Properties of Caffeic Acid

Antioxidants perform by blocking or decreasing the results regulated *via* free radicals and oxidizing compounds (75). The phenolic acids are distinguished *via* the existence of a benzene ring, a carboxylic acid group, and one/more hydroxyl/methoxy groups of the molecule that confers antioxidant functions (76). CA has been revealed for producing defensive results on α-tocopherol in low-density lipoprotein (77, 78). CA is an antioxidant, which may decrease oxidative stress made in the body because of free radicals. Hence, oxidative stress is described as an inequity between the making of ROS and antioxidant protection (79, 80).

In consequence of this inequity, oxidative stress is frequently responded with the progression of several human diseases including cancer (81, 82). Antioxidants perform by inhibiting the effects regulated *via* free radicals and oxidizing compounds (75). CA was found to be an α-tocopherol defensive in LDL (77). Additionally, its grouping with other products, including chlorogenic and caftaric acids, explained high potent antioxidant action in various systems (83, 84). CA is among the hydroxycinnamates used to increase dietary products’ constancy (85, 86). The antioxidant promise of CA is influenced by factors including the nature of oil, food processing, constituents, and the ratio of antioxidants and lipid constituents (86, 87). However, novel dihydro-CA amides were proven to have brilliant free radical scavenging capabilities accessed *via* the DPPH technique; an outstanding potential was identified for protecting polyunsaturated oils (88). The addition of CA was documented to increase the solidity of different oils *via* the blockage of lipid oxidation (85, 87, 89).

UV radiation is the reason for biological alterations, including photoaging, and injures normal lymphocytes, consequential in their death. CA achieves notable consideration as a potential photoprotective agent (90) and also is present in skincare products due to its antioxidant action. Depending on the exposure time, dose, wavelength, and area exposed, the UV radiation may cause premature skin aging, skin burns, skin cell DNA injury, and skin tumor (91, 92). Though lymphocytes were pretreated with CA for 30 minutes while being irradiated with UVB, a photoprotective result of CA was identified in terms of reduced LPO and decreased DNA injury illustrating the viability of lymphocytes (93). Hence, the photoprotective result of CA might be because of GSH metabolism, which displays free radical scavenging. CA was detected for protecting phospholipidic membranes (94, 95) and opposed vitamins C and E depletion by the membrane (96). CA was found to have photoprotective action against UVB both *in vitro* and *in vivo* (97, 98). CA pretreatment causes a significant reduction in g radiation-motivated DNA injury in cultured lymphocytes (97). However, ischemia/reperfusion (I/R) damage may be caused by ROS (99). Reported models might be used to study defensive results of antioxidants administered before the induction of I/R damage (100, 101). CA was proven to have defensive capabilities against I/R injury in rats’ small intestine (99, 102, 103).

### Anti-Inflammatory Properties of Caffeic Acid

**Figure 1:**
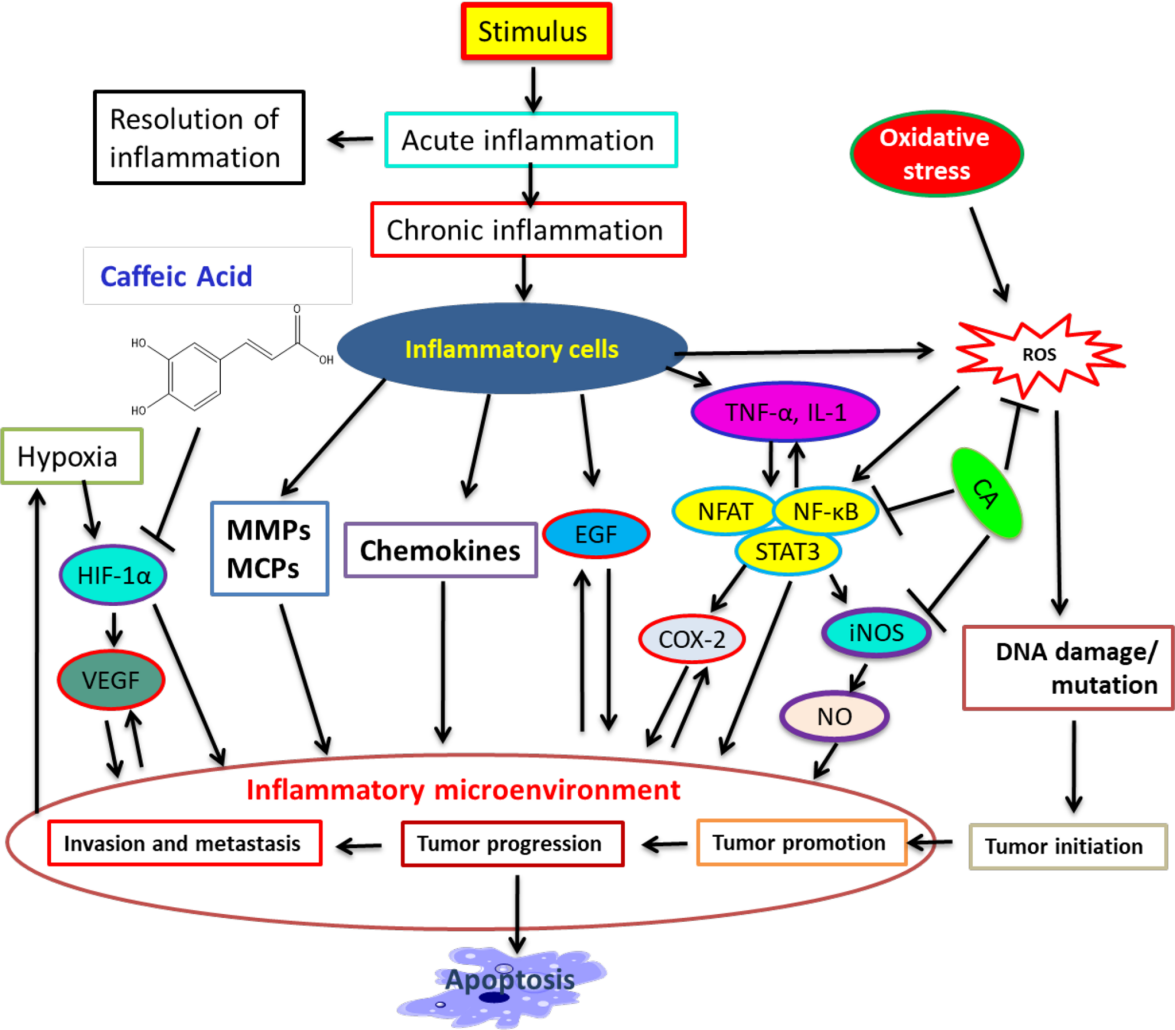
A summary of connection among inflammation, cancer progression, and inhibitory action of CA. Many modulators involve inflammation in tumor progression. CA binds with modulators and represses cancer progression and stimulates apoptosis. [Adapted from Murtaza et al. (115) and Alam et al. (116)].

In response to a stimulus including tissue injury, inflammation expands. It is a physiologic process that might contribute to cancer progression *via* several intermediates (104). Many modulators involve inflammation to tumor progression, including NF-kB, COX-2, TNF-a, IL-6, Nrf2, iNOS, NFAT, and HIF-1α (104–106). CA illustrated its inhibitory action on NO-making that also sturdily blocked the creation of COX -2 and iNOS (107). Therapeutic agents that target NF-κB and COX-2 can help repress cancer angiogenesis (60, 64, 108, 109). CA powerfully hinders ceramide-mediated NF-κB action (110) and UVB-mediated COX-2 expression (109, 111). Hence, numerous studies have recognized CA to be an inducer of cell death in tumor cells and to be able to perform cancer growth blockage and failure in animals (112–114). The role of CA in inflammation, cancer progression, and other pathways is illustrated in **Figure 1**.

However, the activation of cells under pathological conditions was revealed for contributing to the progression of numerous neurodegenerative diseases. The discharge of pro-inflammatory mediators in the brain stimulated through various stimulants, including Aβ, was exhibited to account for the inflammatory constituent of neuronal loss in AD (117–119). CA and its derivatives’ recognized anti-inflammatory result are of prospective benefit in combating AD through these agent candidates.

In addition, activation of Nrf2 has been explained for inhibiting inflammatory gene expression (120, 121) by signaling crosstalk linking the HO-1 (122). Kim et al. (123) have illustrated that Nrf2-induced HO-1 introduction of CAPE is correlated with its anti-inflammatory and antioxidant mechanisms. Hence, there is a relationship between CA derivatives’ antioxidant and anti-inflammatory effect and their therapeutic promise to AD through their multifunctional results. A research (22) has confirmed that the enhanced NF-κB, p65, and 5-LOX correlated with the global cerebral ischemia-reperfusion neuronal injury and memory failure in rats is inverted through CA (10–50 mg/kg) treatment. However, CA (50 mg/kg) also improved neuronal loss and infarct volume 24 h after ischemia (124). The anti-inflammatory result of other CA esters in microglial cells was connected to the induction of HO-1 (105, 125).

## Anticancer Effect of Caffeic Acid

CA has potential anticancer roles in several human cancers (126–128). Literature analysis led to attractive findings on CA and its therapeutic potentials. Several studies explain an inhibitory effect of CA on cell migration and invasion, with potential activity in decreasing metastases in tumor cells (129). Below are the effects of CA on numerous cancers.

### Breast Cancer

One of the deadliest malignancies among women is breast cancer. The migration rate inhibition of breast tumor is treated *via* CA (130). CA decreased MDA-MB-231 and MCF-7 cell growth, repressing breast tumors’ proliferation (131). The experimental and clinical findings reveal a variety of antitumor functions of CA against ER^+^ and ER¯ breast tumors, which can sensitize cancer cells to tamoxifen and decrease breast tumor growth (131, 132). This study illustrates that CA mimics the activities of antiestrogens and modifies main growth regulatory signaling ER/cyclin D1 and IGF-IR/pAkt, resulting in damaged cell-cycle development and decreased cellular proliferation (131). CA exhibits a potent antiradical and antioxidant activity in breast tumors, acting by diverse mechanisms. It is a potential and practical clinical move toward presenting less toxicity for healthy cells and more action toward their malignant counterparts (133). CA decreases IL-12-making and NF-κB activation. However, it hinders with the TLR4 pathway *via* disrupting the TLR4/MD2 complex. Decreased regulation of TRIF, TLR4, and IRAK4 expression provokes apoptosis in breast cancers (10).

### Prostate Cancer

Prostate cancer (PCa) is the most frequent cancer in developed countries (116, 134), resistant to cell death. Due to this resistance, it is necessary to develop a novel therapeutic approach; phenolic acids have revealed a relationship with a reduced risk of PCa (135, 136). CA has been linked with a lesser risk of advanced PCa. However, more intake of CA can be connected with decreased risk of PCa (137). CA treatment reduced Skp2 and Akt1 expression in LNCaP cancers as compared with control (138). NF-κB is constitutively stimulated in PCa cells and inhibition of NF-κB action that links with the repression of invasion, angiogenesis, and metastasis. IκBα inactivates the NF-κB transcription *via* fronting the nuclear signals of NF-κB proteins and keeps them inactive in the cytoplasm (137). CAPE is an NF-κB inhibitor as well as a 5α reductase inhibitor. It is potent for the treatment of PCa. CAPE may block NF-κB activation in PC-3, and it inhibits NF-κB activation (139).

### Lung Cancer

Lung cancer is the leading cause of malignancy-related death worldwide (140). NSCLC is reported to comprise more than 85% of all lung cancers, and approximately 60% of NSCLC presents with advanced stage at the time of diagnosis (140, 141). However, chemotherapy to NSCLC is inadequate because of adverse effects of drug resistance and it contributes small to survival (142). CA may prevent oxidative stress-induced exposure to UVB and DNA injury in lymphocytes (93). A study has illustrated the defensive effect of CA on PTX-mediated apoptosis in A549 cells by the NF-κB pathway (143). Another study has found that a dose of CA (600 μM) drastically stimulates apoptosis in H1299 and A549 cells and has a potential function in modulating and increasing PTX-mediated cell death of NSCLC in *in vivo* and *in vitro* MAPK signaling. However, the combined treatment of CA with PTX reduced the proliferation of H1299 cells except for not normal Beas-2b cells. CA blocked H1299 cell growth *via* stimulating apoptosis, and PTX and CA combined created a synergistic antitumor result in H1299 cells (144). The effects of chemopreventive agents on TGF-β-mediated invasive phenotype utilizing A549 cells as a model scheme have been examined using CAPE as a therapeutic drug. CAPE efficiently repressed TGF-β-increased cell motility and TGF-β-mediated Akt activation and a specific PI3K/Akt signaling inhibitor, LY294002 (145, 146).

### Melanoma

Because of the strong resistance of melanoma to conventional chemotherapy, numerous studies focused on novel treatment approaches and coadjuvants were investigated. Studies representing an inhibitory effect of CA on cell migration and invasion with potential activity in decreasing metastases in tumor cells are attracting more attention and are increasingly frequent in the scientific data (129). Additionally, research explains the potential of CA in decreasing proliferation, growth, and cell viability in numerous cancers, and a capability to induce *via* apoptosis (129, 147). Results illustrated a reduction in cell viability, inhibition of colony formation, cell cycle modulation, and modifications in the gene expression of caspases following CA treatment (148). However, these results recommend an anticancer effect of CA on SK-Mel-28 cells. CA might play a key function in inhibiting cancer progression in melanoma cells (148) and efficiently blocked melanin-making in B16 melanoma cells (149). However, CAPE is recommended for suppressing ROS-mediated DNA damage in A2058 cells compared to other potential defensive agents (150).

### Oral Cancer

Oral cancer is the most general kind of head and neck cancer. Hence, more than 90% of oral cancers are OSCC (151–153). Pro- and anti-apoptotic Bcl-2 proteins are involved in oral cancer (154–156). The migration rate of OSCC may be drastically blocked *via* the biological action of CA (157). Treatment with CAPE prevents the expression and activity of COX-2 in OSCC (158). CAPE treatment can inhibit the activity and plenty of COX-2 and EGFR. Administration of CAPE may inhibit and hinder the progression of oral tumors (159). Hence, CAPE is sturdily efficient against oral cancer cells. It prevents the COX-2 and EGFR activities and proliferation, growth, survival, and metastasis, and it hampers with the PI3K-Akt pathway and Skp2 in oral tumor (159).

### Liver Cancer

HCC is a leading form of liver cancer, distinguished by being a malignant main solid tumor that differs from hepatocytes (160, 161). CA performs in HCC *via* its strong antioxidant capability, which inhibits ROS-making, decreases oxidative stress, and is very general in liver cancer (162, 163). CA performs as a primary as well as secondary antioxidant. It may act in the angiogenesis of HCC cells by decreasing the phosphorylation of JNK-1 and reducing the activation of HIF-1α. Animal studies treated with CA and CAPE proved the endorsed repression of cancer growth in HepG2 and the reduction of cancer invasion on a metastatic position in the liver. However, CA and CAPE prevent and obstruct the MMP-9 action *via* blocking NF-κB function (11, 164). *In vitro* and *in vivo* investigations have revealed that CA exerted its antihepatocarcinoma result, dependent on a variety of mechanisms including cell death *via* induction of TRAIL signaling and the activation of caspase 9, liberation of *cyt-c*, and creation of the apoptosome (32). Hence, CA is an anticancer agent for inhibiting HCC (165). CA was established to be a stimulant of HO-1, GCLC, and GCLM expression by the Nrf2/ERK pathway, and it can be an efficient chemoprotective agent to defend liver injury against oxidative harm (166).

### Cervical Cancer

CA has been known for its antioxidant function in normal cells and pro-oxidant function in tumor cells. Hence, this pro-oxidant-induced oxidative DNA break and its downstream pathway stimulate cancer cell death (167, 168). CA treatment has altered ROS and modified MMP in ME-180 and HeLa cells (167, 169). The enhanced apoptotic morphological alterations in CA-treated cells were examined in ME-180 and HeLa cells. CA possesses an antitumor effect *via* its pro-oxidant function. It considerably decreases the proliferation of HeLa cells in a concentration-dependent manner. The morphological proof of cell death such as nuclei fragmentation has been noticeably detected after exposure to CA by flow cytometry. CA reduces the levels of uncleaved caspase-3 and Bcl-2 and provokes cleaved caspase-3 and p53 (170).

CA stimulates apoptosis *via* blocking Bcl-2 action, leading to the liberation of cyt*-c* and following caspase-3 activation, representing the fact that CA induces cell death by the mitochondrial apoptotic pathway. CA has a potent anticancer effect and can be a promising chemotherapeutic agent (170). It interrelates synergistically with 5−FU, leading to a decline of apoptosis in HeLa cells with the lowest quantity of hemolytic activity (171). CDDP and CA have been selected, and their CxCa antitumor action has been calculated in combination. In CaSki and HeLa cells, a combination index <1 to CDDP and CFC showed the synergistic growth/survival inhibition that drastically enhanced caspase−3, −7, and −9 expression. CFC can be an aspirant antitumor agent and, when exploited in combination, might enhance the therapeutic efficiency of CDDP (172). Synergistic results of CA-cisplatin stimulate apoptosis in cervical cancer cells by the mitochondrial pathway (172).

### Colorectal Cancer

Colorectal cancer (CRC) is the third leading cause of malignancy-related death in the United States. Huge common CRC cases may be attributed to environmental reasons, as they account for more than 70% of all occurrences (173). Some features of western dietary outlines can also persuade the risk of CRC. However, adiposity and high insulin levels in obese subjects are associated with an enhanced risk of CRC (174). CA has been revealed to have potential antitumor effects in cell cultures and animal models and might play a defensive role against CRC (175). CA prevents the growth of colon cancer and is a potent antitumor agent that enhances AMPK activation and induces apoptosis in CRC cells. However, the structure of CA may be utilized for the rational plan of novel inhibitors, which target human CRC (176). A meta-analysis documented a decreased occurrence of colon cancer in more versus no- or less-coffee-taking subjects in case-control investigations, but not in cohort studies to CRC (177). CA consumption decreases the risk of colon cancer. It sturdily repressed MEK1 and TOPK actions and bound to either MEK1 or TOPK. CA blocked ERKs phosphorylation, AP-1, and NF-κB and consequently prevented TPA-, EGF-, and H-Ras-mediated neoplastic transformation of JB6 P+ cells. CA targets MEK1 and TOPK for suppressing colon cancer metastasis as well as neoplastic cell transformation (15). It endorses apoptosis in HCT-116 and SW-480 in a dose-dependent way (176). Treatment with CA inhibited PDK1, Akt, and mTOR phosphorylation.

### CA Prevents Photoaging and Photodamage of the Skin

CA-blocks UVB-irradiation mediated more expression of MMP-1 and -9 in human skin fibroblasts. CA-affected abdominal skin repressed UVA-irradiation-mediated ROS; CA was found in the skin after oral ingestion (178). CA pretreatment decreased the cytotoxicity of HaCaT after UVA irradiation and repressed UVA-irradiation-mediated MMP-1 action and mRNA oxidant creation. CA upregulated the GSH content and mRNA of γglutamate-cysteine ligase, and mRNA catalase and glutathione peroxidase expression in UVA-irradiated cells. CA generated defensive effects on UVA-irradiation-induced MMP-1 induction in HaCaT, probably as it restored the antioxidant protection system on cellular and molecular levels (178, 179). However, CA showed weak collagenolytic action; topically concerned CA defended the skin from UVB-irradiation-mediated erythema. *In vitro* and *in vivo* research revealed that CA can be effectively utilized as a topical defensive agent against UV-irradiation-mediated skin injury (178).

CA attenuates the local immune repression provoked *via* UVB irradiation and blocks the UVB-irradiation-mediated IL-10 promoter, IL-10 mRNA expression, and protein-making in mouse skin (178, 180). CA can present IL-10-making by interfering with prostaglandin E2 production connected to stimulating the UVB-irradiation-mediated immune repressive cytokine system. CA considerably prevented the UVB-irradiation-mediated activation of MAPK pathway AP-1 and NF-κB. It may have a therapeutic potential as a topical defensive agent against the adverse effects of UVB radiation (181, 182). CA blocked the TPA-dependent motivation of DNA synthesis and enhanced the number of cancers. CA efficiently suppressed UVB-irradiation-mediated COX-2 and prostaglandin E2 in JB6 cells and repressed UVB-irradiation-mediated skin carcinogenesis *via* preventing the Fyn kinase action (178, 183).

### Caffeic Acid Combined With Chemotherapeutics or Nanoparticles

The combination therapy is the prevention and treatment strategy with two or more drugs with the target of accomplishing the comparable effectiveness levels with negligible toxicities at doses lesser than normal, which induces better synergistic/additive effects (184, 185). Hence, successful combinations of promising therapeutic drugs/nanoparticles with products can achieve the needed conclusion but with a slighter toxicity outline (186, 187). CA and cisplatin illustrated potent antitumor action in the tumor. Furthermore, cisplatin-sensitive cells, while exposed to combination therapy of CA and cisplatin, which elucidated speedily, improve in the activity of apoptotic cascade by increased caspase action in contrast to only administrating 5 μM cisplatin. However, combining 5:50 μM (cisplatin/CA) enhances the caspase activity *via* 4:3 folds through 60% cell viability in A2780cisR cells (188). However, cisplatin combined with CA improved its therapeutic consequence that led to the inhibition of cell survival of HeLa and CaSki cells, which could be revealed by the synergistic effect. Therefore, this combination was correlated with increasing caspase-3, -7, and -9 (172).

Metformin (Met) and CA were recognized to have synergistic results, while combined with anticancer therapies, chiefly to HTB-34 cells (126). CA combined with Met and cytotoxicity mechanism moved toward apoptosis without distressing healthy human fibroblasts. Studies continue to examine the mechanism of the anticancer role of the combination of Met and CA (189). The cytotoxicity of CA-coated iron oxide nanoparticles (CAMNPs) enhanced twofold in tumor cells. The proficient features of CAMNPs may be integrated into appliances, therefore enhancing the efficiency of clinical tumor treatment (190). Antitumor action of nanoparticles has been evaluated with DOX-resistant CT26 cells. Hence, DOX-loaded nanoparticles of ChitoCFA/CMD are a potent vehicle for antitumor drug targeting (191). The CA-SLN formulation might be potent as an alternative nanosize-based drug delivery system for tumor treatment (192).

## Neuroprotective Effect of Caffeic Acid

CA generates antidepressive-like action in forced-swim mice (19, 193). It demonstrates anxiolytic-like results in rats running a maze, which defends against H_2_O_2_-mediated oxidative break of rat brain tissue (19, 194). CA attenuates the less regulation of the brain-originated neurotrophic factor that plays significant functions in depression pathophysiology and treatment (195, 196). It decreases pathological states after focal cerebral ischemia in rats, which attenuates neuronal injury, astrogliosis, and glial scar-making in cryoinjured mouse brains (19). CA decreases oxidative stress in the rat hippocampus following pilocarpine-mediated seizures (197) and defends mouse brains from aluminum-mediated injury (198). It decreases inflammatory damage in the striatum of MPTP-treated mice (199, 200). CA defends cerebellar granule neurons from cell death mediated *via* the neurotoxin MPP+ (201). It illustrates neuroprotective results against amyloid-β–mediated neurotoxicity *via* blocking calcium influx as well as tau phosphorylation (202). CA can contribute to inhibiting neurodegeneration and brain damage, and articulates anxiolytic-like functions. Systemic administration of LPS mediated profound immobility, enhanced the TNF-α and IL-6 levels, and changed the host antioxidant protection in animals (193). Several *in vitro* studies revealed that CA exhibits a broad-spectrum neuroprotective summary. CA is defensive against the H_2_O_2_-mediated oxidative stress that attenuated the H_2_O_2_-mediated cell damage in cultured cerebellar granule neurons (203). CA decreased the acrolein-mediated neurotoxicity *via* the Akt/GSK3 pathway stimulation in HT22 cells (73). *In vitro* studies demonstrate that CA defends neurons from a broad range of apoptosis-mediating agents. A study (204) proved that repetitive administration of CA defended the mouse brain from aluminum-mediated injury. CA can have particular selectivity related to the AChE from diverse brain regions (205). Hence, statistics from animal studies show that CA might inhibit neuronal injury/death caused *via* diverse stressors saying that CA is a potent neuroprotective agent for the treatment and prevention of neurodegenerative diseases **(** **Table 1****).**

**Table 1:** Summary of *in vivo* studies on the neuroprotective effects of caffeic acid.

Animals	Model	Treatment	Main Outcomes	Ref.
Male Wistar rats	Aluminum-mediated neurotoxicity	100 mg/kg (p.o.)—11 days	(1) Improved memory; (2) decreased AChE, CAT, and GST action; (3) decreased GSH and nitrite levels	(206)
Wistar rats		10–100 mg/kg (p.o.)—30 days	(1) Recovered learning and memory; (2) reduced AChE action in cerebral cortex and striatum; and (3) enhanced AChE action in the cerebellum, hippocampus, and hypothalamus	(205)
Wistar rats	Streptozotocin-mediated dementia	10–40 mg/kg (p.o.)—21 days	(1) Attenuation of the streptozotocin-mediated learning and memory destructions; (2) enhancement in AChE action; (3) enhancement in MDA, nitrite, and protein carbonyl levels; and (4) reduction in the GSH level	(207)
Wistar rats	Pilocarpine-mediated seizures	4 mg/kg (i.p.) 30 min before pilocarpine injection	(1) Anticonvulsant-like effect, (2) reduced LPO level and nitrite content, (3) enhanced SOD and CAT action	(197)
Fisher rats	Kainic acid-mediated neurotoxicity	50 mg/kg (i.p.) 4 injections	(1) Extended latency to seizures, (2) decreased neuronal loss in CA3 hippocampal field	(208)
Male Wistar rats	Quinolinic acid-mediated neurotoxicity	5 and 10 mg/kg (p.o.)—21 days	(1) Enhancement of locomotor action and motor coordination, (2) repaired redox status in the striatum	(209)
CF1 mice	Pilocarpine- and pentylenetetrazole-mediated seizures	4 and 8 mg/kg (i.p.) 30 min before seizure induction	(1) No anticonvulsant-like effect, (2) defense against pilocarpine-mediated genotoxic injury in the hippocampus	(210)
Sprague–Dawley rats	Focal cerebral ischemia/reperfusion injury	50 mg/kg (i.p.) 30 min before ischemia induction and 0, 1, 2 h	(1) Decline of neurological deficits, (2) reduced neuron loss, infarct volume, brain atrophy, and astrocyte proliferation, (3) blockage of leukotriene making	(124)
Sprague–Dawley rats	Cerebral ischemia/reperfusion injury	50 mg/kg (i.p.) instantly after ischemia induction and then frequently for 12 h	(1) Enhanced neurological deficit scores, (2) decreased infraction volume, (3) reduced 5-LOX	(211)
C57BL/6J mice	Rotenone-mediated neurotoxicity	50 mg/kg (p.o.) for 1 week before rotenone exposure, and then 5 days	(1) Inhibited degeneration of dopaminergic neurons in substantia nigra, (2) more regulated metallothionein-1 and 2 in striatal astrocytes	(212)
C57BL/6 mice	MPTP-mediated neurotoxicity	0.5–2% in the diet—4 weeks	(1) Reduced inflammatory cytokines levels; (2) repressed NO, prostaglandin E2, and GFAP making; (3) preserved BDNF, GDNF, and tyrosine hydroxylase levels; (4) better synthesis of dopamine	(200)
Sprague–Dawley rats	LPS-mediated neurotoxicity	50 mg/kg (p.o.) 10.5, 5.5, and 0.5 h before LPS injection	Attenuation of LPS-mediated failure of dopaminergic neurons and microglial activation in the substantia nigra	(213)

Neurodegenerative disorders include a heterogeneous cluster of diseases linked to progressive deterioration of the structure and performance of the peripheral or CNS. The categorization of neurodegenerative diseases depends on clinical symptoms, damaged brain areas, influenced cell types, changed proteins, and etiology. However, affected brain areas have symbols of atrophy and defective metabolic action (214). CA affects Parkinson’s (215, 216) and Alzheimer’s diseases and other neurodegenerative diseases (216, 217)

### Alzheimer’s Disease

Alzheimer’s disease (AD) is a progressive, neurodegenerative disorder distinguished *via* deterioration of cognition and memory, progressively interfering with daily living actions attended through neuropsychiatric symptoms and behavioral disorders (121, 218). However, AD is the most general form of dementia (219). The disease is distinguished by general behavioral symptoms such as apraxia, agnosia, aphasia, erratic emotion, sleep disorders, and interpersonal or social worsening (220, 221). One of the well-identified pathological characteristics of AD is the deposition of amyloid-beta (Aβ), which contributes to neuronal cell death by several mechanisms, such as the disorder of cellular oxidant–antioxidant equilibrium. However, the agent can modify the Aβ creation that increases the antioxidant in neuronal cells, which increases cell viability and might offer therapeutic opportunities for AD (222). CA administration affected a considerable reduction in AChE action and nitrite production in rats with AD compared with the AD model (223). CA repressed inflammation, oxidative stress, NF−κB−p65 protein, and caspase−3 action and regulated the p53 protein expression and p-p38-MAPK expression in rats with AD. However, the useful effects of CA on learning deficits in the AD model were because of the repression of inflammation and oxidative stress by the p38-MAPK pathway (223). CA inhibited AlCl_3_-mediated dementia in rats (206). It blocked memory deficits mediated through the focal cerebral ischemia (224). The expression of p-tau protein reduced in the rat’s hippocampus administered with CA in contrast to the HF group (225). The antidementia action of CA against AlCl_3_-mediated dementia in rats (206). Hence, the conclusion of this study suggests a wider extent for screening CA against neurodegeneration-linked disorders.

**Figure 2:**
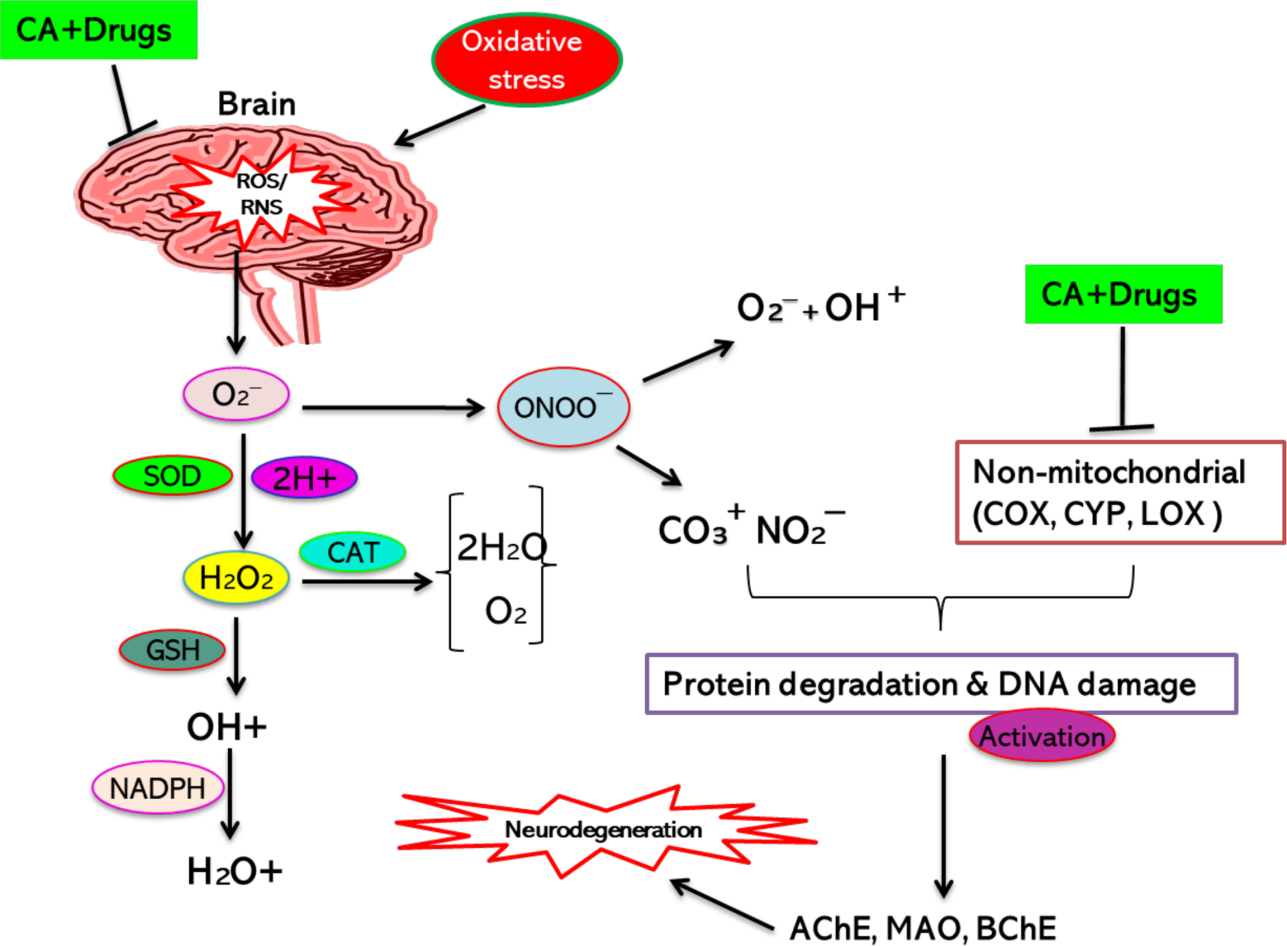
CA’s synergistic action with other agents/drugs (neuroprotective) for inhibiting neurodegeneration. [Adapted from Maity et al. (228)]. This figure was drown by ChemBioDraw.

The combined result of CA and ChA against AD has been performed to find its synergistic/additive result in the biological scheme. The approach targeting the inhibition of AChE and BChE has an excellent effect against AD (226). The combination therapy of ChA and CA demonstrated a synergistic action. However, the mechanism by which the combination therapy exerts its neuroprotective functions is to prevent AChE and BChE actions and inhibit oxidative stress-mediated neurodegeneration (227). The detected synergistic result of combination for these enzymes may improve efficiency and protect these ingredients against neurological states, including AD and PD **(** **Figure 2****).** CA and CAF combination treatment on a specific ratio might be an enhanced option for further assessment against AD. However, the food ingredients are found in combination and hence promising for exerting a superior neuroprotective effect (229).

### Parkinson’s Disease

Parkinson’s disease (PD) is a progressive and degenerative disease that affects more than 6 million public (230). Motor, as well as nonmotor, symptoms manifest it. Hence, the motor symptoms include resting tremor, bradykinesia, rigidity, and postural instability. Spontaneous movement is drastically reduced, with the failure of facial expression, decreased blink rate, and damaged spontaneous swallowing, which affects sialorrhea. However, patients with PD can experience retropulsion and festination (231, 232). The nonmotor symptoms include anxiety, dysautonomia, cognitive decline, anosmia, depression, urinary complaints, disturbances, sleep, and orthostatic hypotension (233, 234). CA’s inhibitory action against α-synuclein fibrillation could lead us to plan novel therapeutic agents for PD (235). CA pre-intake has sustained TH action, protein-making, and dopamine synthesis. It is a promising neuroprotective drug for the development of PD (236). CA possesses neurotrophic results exerted by the modulation of Akt and ERK1/2 pathways, hence representing potential agents for finding neurotrophic compounds for the treatment of neurodegenerative disorders (237). However, the anti-inflammatory action of CA emphasized its neuroprotective action against rotenone-mediated neurodegeneration in mice (238).

## Biomolecular Interactions

CA interacts with several protein molecules, genes, and chemicals. Around 9 enzymes are identified with similarity >0.85 that CA targets. **Table 2** represents the list of target proteins generated from The Binding Database (http://www.bindingdb.org).

**Table 2:** List of enzyme inhibition constant data from Binding DB for compound caffeic acid similarity at least >0.85.

Target name	Max similarity	Hits
β-Carbonic anhydrase 2 (CA 2)	1.00	1
β-Carbonic anhydrase 3 (CA 3)	0.88	3
72 kDa type IV collagenase	1.00	1
Aldose reductase	0.85	1
Carbonic anhydrase 2	0.85	1
Catechol O-methyltransferase	1.00	1
Epidermal growth factor receptor	1.00	1
Interstitial collagenase	1.00	1
Matrix metalloproteinase-9	1.00	1

β-Carbonic anhydrase 3 (CA 3) from Mycobacterium tuberculosis is the best target enzyme with a similarity of 0.88 to the ligand. It has the highest negative ΔG° at -8.51 kcal/mol among all the targets. It is followed by β-carbonic anhydrase 2 (CA 2) from Mycobacterium tuberculosis with ΔG° at -8.49 kcal/mol and similarity of 1.00. The PDB includes 12 PDB complex structures with CA. Each is defined in **Table 3** with inferences on inhibitory and activation capabilities of CA.

**Table 3:** List of protein-bound 3D structures complexed with caffeic acid and inferences on binding affinity.

PDB code	Structure description	Inference
1KOU	Photoactive yellow protein complexed with CA	CA binds to the protein in strained conformation, resulting in faster ejection
6I72	Fragaria ananassa O-methyltransferase and S-adenosylhomocysteine complexed with CA	–
6YRI	HCAII complexed with CA	catechols as inhibitors for CA
4YU7	Piratoxin I complexed with CA	CA neutralized myotoxic activity of PrTX-I (snake venom)
3S2Z	*Lactobacillus johnsonii* cinnamoyl esterase LJ0536 S106A mutant in complexed with CA	–
4N0S	ERK2 complexed with CA	Yang et al. (23) suggested CA targets and inhibits ERK1 and 2 for cancer prevention against SUV- induced skin cancer
4FB4	ABC-transporter family protein complexed with CA	–
2O7D	Tyrosine ammonia-lyase complexed with caffeate	–
3HOF	Macrophage migration inhibitory factor (MIF) complexed with CA	–
4EYQ	ABC transporter HaA2 in complex with CA/3-(4-hydroxy-phenyl) pyruvic acid	–
5VFJ	BnSP-7 complexed with CA	CA as the best inhibitor for MDoS region of BnSP-7
6AWU	PR 10 Allergen Ara h 8.01 in complex with CA	–

**Figure 3:**
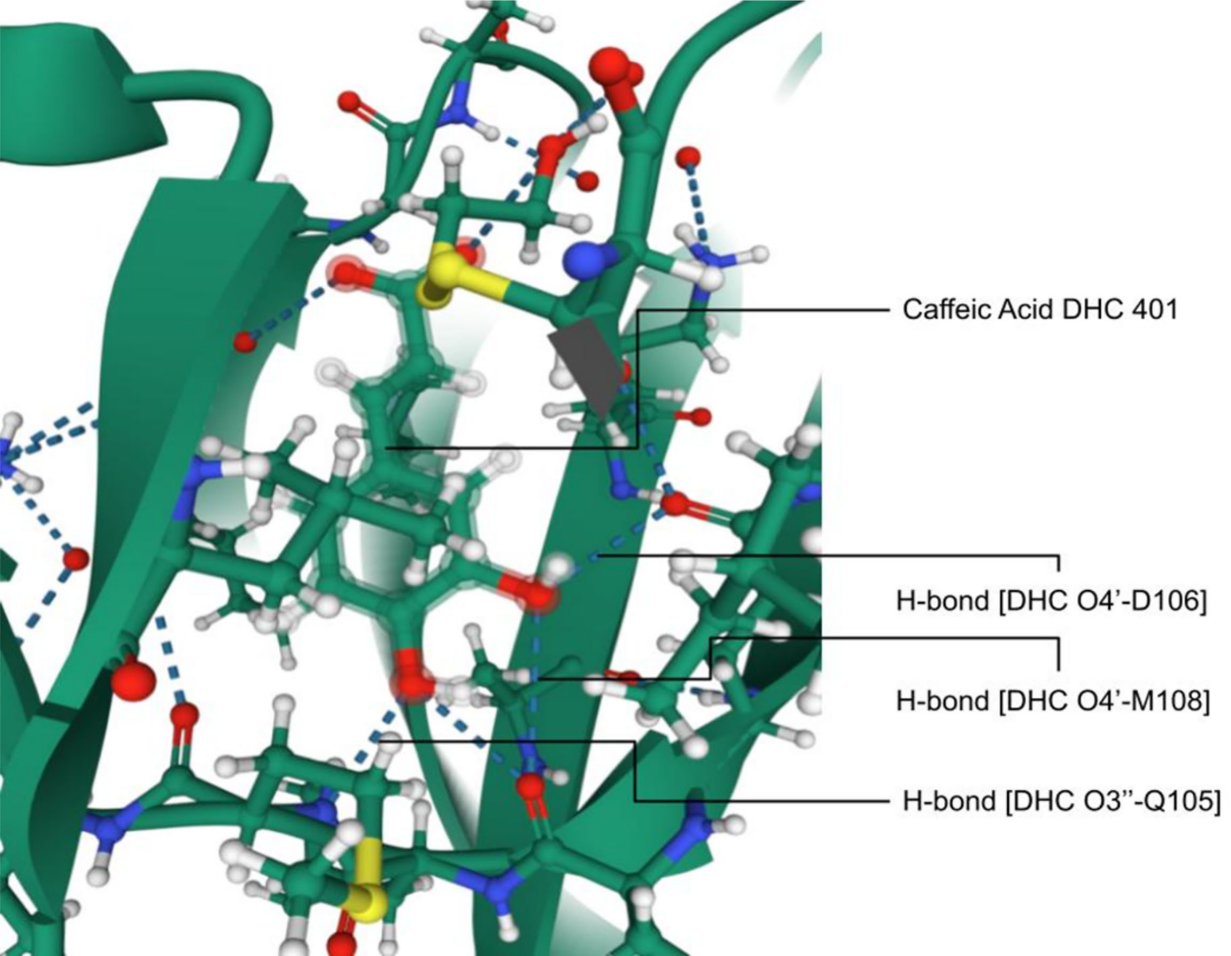
Interaction of ERK2 with caffeic acid; hydrogen bonding between DHC O4’-D106, M108, and CA O3’-Q105 imparting molecular level inhibition of the protein [Adapted from Yang et al. (23)].

The PDB ID 4N0S, ERK2 complexed with CA has potential medical significance with validation. In a study on ERKs, CA binds strongly with ATP-binding cleft through hydrogen bond formation to specific amino acids (**Figure 3**). The residues on the hinge loop, Q105, D106, and M108, bind to the 3’ hydroxyl and 4’ hydroxyl ends, respectively (23), thus proving the molecular level impact of CA on enzymes. The experiments were performed *in vivo* in mice. CA suppresses the SUV-induced ERK phosphorylation with further downstream signaling in HaCaT cells. CA reduces SUV-induced skin cancer before or after exposure to SUV (23, 239). Studies also revealed that the knockdown of ERK2 reduced CA sensitivity in skin cancer cells. Reduction in ERK2 levels decreases CA’s ability to prevent skin cancer. CA suppresses skin cancer when exposed to SUV in mice. It is an inhibitor of ERK1/2 and blocks the activities of downstream substrates such as Elk1, c-Myc, and RSK2. However, CA may be a good anti-skin cancer agent and effective against SUV-mediated skin tumors (23, 239, 240).

## Molecular Targets of Caffeic Acid in Therapeutics

CA attaches directly with Fyn noncompetitively by ATP and represses UVB-mediated COX-2 and PGE2-making *via* the inhibition of Fyn kinase action *in vivo* (241, 242). The inhibition affected the attenuation of UVB-mediated COX-2 promoter action and AP-1 and NF-κB in JB6 P+ cells. UVB-mediated phosphorylation of JNKs, p38, and ERKs is found in JB6 P+ cells (243, 244). CA is more efficient than chlorogenic acid at preventing Fyn kinase and in repressing the activation of MAPK pathways. However, B-Raf mutations happen commonly in colon polyps and are an early starting incident in melanoma and colon cancer (245). MEK1/2 has unique features among the components of MAPK pathways (246, 247).

**Figure 4:**
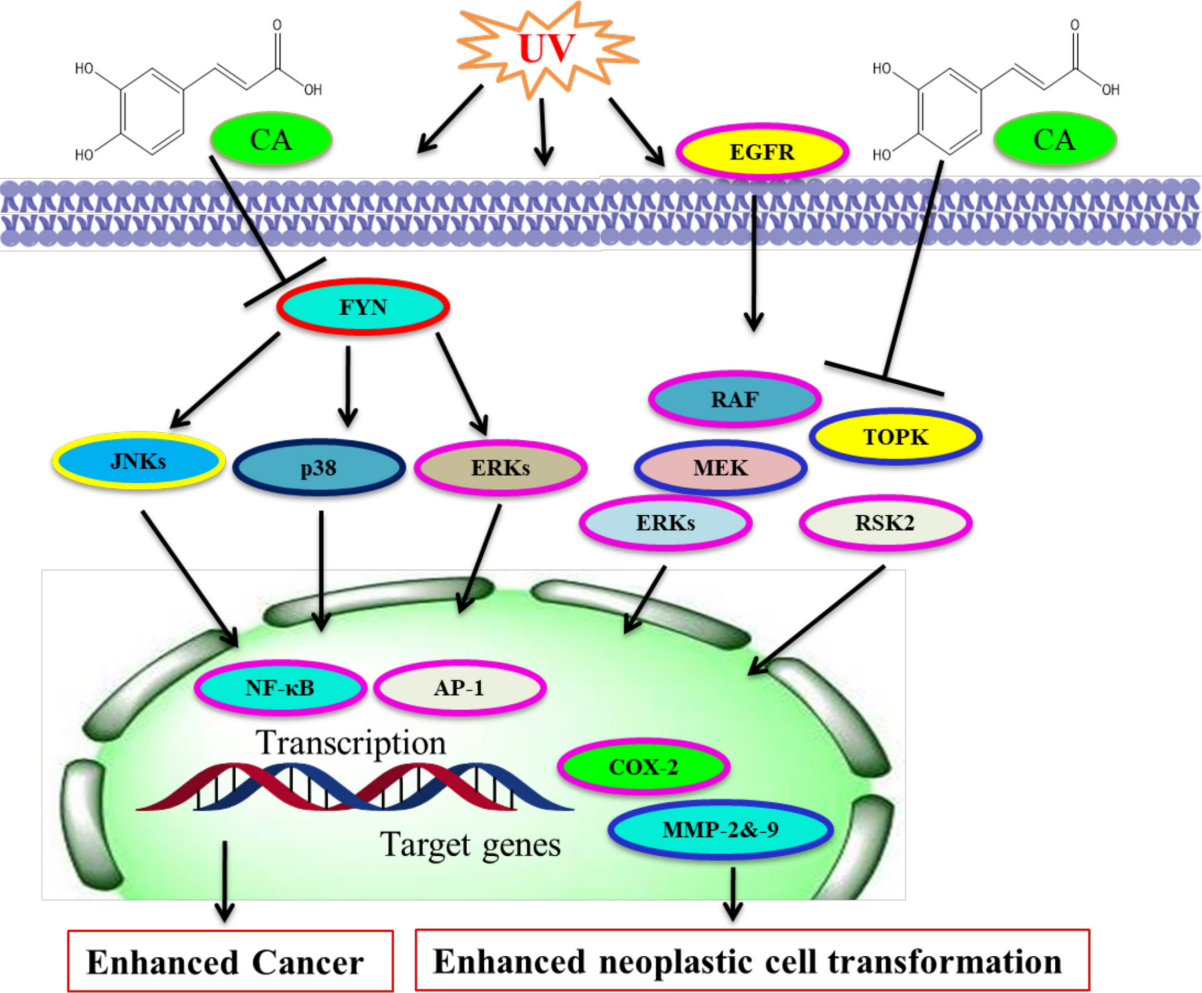
Molecular targets of caffeic acid. CA binds with Fyn for blocking its kinase activity and UV-mediated skin cancer. CA binds with MEK and TOPK to prevent their kinase actions and neoplastic cell transformation. [Adapted from Bode et al. (245)]. This figure was drown by ChemBioDraw.

Constitutive MEK1 activation affects transformation and cancer, whereas an inhibitor of MEKs represses transformation and cancer growth in mouse models and cell culture (248, 249). Lymphokine-activated killer TOPK is a kinase; an activator of ERKs and TOPK action emerges to be engaged in TOPK’s oncogenic role in tumors (245, 250). CA prevented CT-26 cell-mediated mouse lung metastasis in mice and neoplastic cell transformation through repressing ERKs phosphorylation (15, 245). Decaffeinated coffee or CA prevented CT-26 cell-mediated COX-2, MMP-2, and -9 action, and ERKs phosphorylation (251, 252). Computational modeling study results together with laboratory experiments exhibited that CA interacted particularly with mitogen-activated MEK1 and TOPK with ATP and repressed their respective kinase action (245). The inhibition of TOPK and MEK1 has been linked with TPA-mediated ERKs and p90 RSK2 and attenuation of AP-1 and NF-κB transactivation (245, 253, 254). CA/coffee consumption has been linked with reduced ERKs phosphorylation in colon cancer (242, 245, 255). However, the molecular targets and CA effects are summarized **(** **Figure 4****).** CA was confirmed to be a stimulant of HO-1, GCLC, and GCLM by ERK/Nrf2 signaling, and it could be an efficient chemoprotective drug for defending liver injury against oxidative damage (166). CA exerts chemopreventive action against solar UV-mediated skin carcinogenesis *via* targeting ERK1/2 (23).

Some pharmacological effects of CA on CNS, with/without the initiation of neurotoxic results, have been elicited by a molecular target. The treatment with CA has detected a defensive effect against H_2_O_2_-mediated oxidative injury on brain tissue. Hence, CA stimulated DNA breaks in brain tissue (256). CA’s neuroprotective results against Aβ-mediated neurotoxicity *in vitro* prevent peroxynitrite-mediated neuronal injury (257, 258). CA performs as a selective 5-LOX inhibitor and defended mice from aluminum-mediated neuronal injury by the downregulation of APP and Aβ protein (204). The antioxidant effect of CA has been explained to be neuron-defensive *in vivo* and ameliorates brain damages after focal cerebral ischemia in pathological states (124). CA and its interface with intracellular molecules tell that CGA exerts its neuroprotection through its caffeoyl acid set, potentially being a therapeutic drug in neurodegenerative diseases linked with glutamate excitotoxicity (259). A natural and potent CA might be considered promising therapeutic in diseases that link the cholinergic system (205).

## Conclusion and Future Directions

CA, a phenolic compound, is plentiful in medicinal plants. It has numerous biological effects, including antioxidation, anti-inflammation, anticancer, and neuroprotective. CA is involved in several biochemical pathways and suitable targets are also identified. The biological activities of CA come from its functional groups. It is possible to enhance the anticancer capabilities of CA by altering these functional sites. The potential therapeutic effects of CA are mediated *via* repression of MMP-2 and -9 and inhibited NF-κB, AP-1, ERKs, STAT3, and VEGF. The activity of CA is displayed in several molecular mechanisms that regulate cell survival and stimulate apoptosis.

Broad *in vitro* and *in vivo* examinations have revealed that CA exerts anticancer and neuroprotective effects indicating its preventive and therapeutic potential for diverse cancers and neurodegenerative disorders. Hence, the multifunctional properties of CA have been illustrated for displaying the anti-AD result *in vitro* and *in vivo*. The diversity of biochemical mechanisms of CA was exhibited as a promising therapeutic potential for neurological diseases. The effects of CA in cancer and neurodegenerative disorders could be affected by the synergistic activity of various active agents. The therapeutic effects of CA may be synergistically increased by other drugs/agents. However, the clinical study must be persisted to entirely evaluate the relationship between CA and other agents/drugs and the risk of cancer and neurodegenerative diseases.

Further studies, the basic research, clinical study, and epidemiological level, carried out with a standardized strategy are required for better understanding the assessment of a broad range of CA in the clinical management and treatment of cancer and neurological diseases. Hence, the inclusive study of CA based on clinical trials could be extremely beneficial in the approach and plan of novel therapies for cancer and neurological diseases.

## Author Contributions

MaA: Conceptualization, writing—original draft preparation, data curation, investigation, and methodology. SaA: Data analysis, validation, and visualization. AME: Formal analysis, supervision, investigation, and validation. MoA: Data curation, validation, and writing—review and editing. ShA: Methodology and writing—review and editing. MIH: Conceptualization, writing—original draft preparation, and investigation. ViP: Conceptualization, data analysis, validation, project administration, writing—review, and editing. All authors contributed to the article and approved the submitted version.

## Funding

This work is supported and funded through the Indian Council of Medical Research (Grant No. 45/6/2020-DDI/BMS).

## Conflict of Interest

The authors declare that the research was conducted in the absence of any commercial or financial relationships that could be construed as a potential conflict of interest.

## Publisher’s Note

All claims expressed in this article are solely those of the authors and do not necessarily represent those of their affiliated organizations, or those of the publisher, the editors and the reviewers. Any product that may be evaluated in this article, or claim that may be made by its manufacturer, is not guaranteed or endorsed by the publisher.

## Abbreviations

CA, Caffeic acid; CAPE, Caffeic acid phenethyl ester; PhA, Phenolic acids; MA, Monocarboxylic acid; OSCC, Oral squamous cell carcinoma; HCC, Hepatocellular carcinoma; COX-2, Cyclooxygenase 2; HCs, Hydroxycinnamic acids; CGA, Chlorogenic acid; AAA, Aromatic amino acids; PLP, Pyridoxal 5-phosphate; NAD, Nicotinamide adenine dinucleotide; TAL, Tyrosine ammonium lyase; NF-κB, Nuclear factor kappa B; VEGF, Vascular endothelial growth factor; PKC, Kinase C protein; CGA, Chlorogenic acid; JNK, C-Jun N-terminal kinase; MCT, Monocarboxylic acid transporters; CLL, Chronic lymphocytic leukemia; ROS, Reactive oxygen species; H_2_O_2_, Hydrogen peroxide; TNF, Tumor necrosis factor; AChE, Acetylcholinesterase; BChE, Butyrylcholinesterase; STAT3, Transcription factor and signal translation 3; EGFR, Epidermal growth factor receptor; Nrf2, Nuclear factor-E2 p45-related factor; TOPK, T-cell-originated protein kinase; MMP, Matrix metalloproteinases; VEGF, Vascular endothelial growth factor; ECM, Extracellular matrix; ERK, Extracellular signal-regulated kinase
